# Anterior knee pain in younger adults as a precursor to subsequent patellofemoral osteoarthritis: a systematic review

**DOI:** 10.1186/1471-2474-11-201

**Published:** 2010-09-09

**Authors:** Martin J Thomas, Laurence Wood, James Selfe, George Peat

**Affiliations:** 1 Arthritis Research UK Primary Care Centre, Primary Care Sciences, Keele University, Keele, Staffordshire, ST5 5BG, UK; 2 School of Public Health and Clinical Sciences, University of Central Lancashire, Preston, PR1 2HE, UK

## Abstract

**Background:**

Patellofemoral osteoarthritis (PFOA) is a common form of knee OA in middle and older age, but its relation to PF disorders and symptoms earlier in life is unclear. Our aim was to conduct a systematic review to investigate the strength of evidence for an association between anterior knee pain (AKP) in younger adults and subsequent PFOA.

**Methods:**

The search strategy included electronic databases (Pubmed, EMBASE, AMED, CINAHL, Cochrane, PEDro, SportDiscus: inception to December 2009), reference lists of potentially eligible studies and selected reviews. Full text articles in any language, - identified via English titles and abstracts, were included if they were retrospective or prospective in design and contained quantitative data regarding structural changes indicative of PFOA, incident to original idiopathic AKP. Eligibility criteria were applied to titles, abstracts and full-texts by two independent reviewers. Data extraction included study location, design, date, sampling procedure, sample characteristics, AKP/PFOA definitions, follow-up duration and rate, and main findings. Foreign language articles were translated into English prior to examination.

**Results:**

Seven articles satisfied eligibility (5 English, 2 German). Only one case-control study directly investigated a link between PFOA and prior AKP, providing level 3b evidence in favour of an association (OR 4.4; 95%CI 1.8, 10.6). Rough estimates of the annual risk of PFOA from the remaining six small, uncontrolled, observational studies (mean follow-up range: 5.7 to 23 years) ranged from 0% to 3.4%. This was not the primary aim of these studies, and limitations in design and methodology mean this data should be interpreted with caution.

**Conclusions:**

There is a paucity of high-quality evidence reporting a link between AKP and PFOA. Further, well-designed cohort studies may be able to fill this evidence gap.

## Background

Patellofemoral osteoarthritis (PFOA) is increasingly recognised to be a common, early form of knee OA in middle and old age, associated with symptoms and functional limitation [1]. While OA research tends to focus on this older age group, our understanding of the aetiology of PFOA may benefit from taking a broader view of events, exposures, morphology, and morbidity related to the patellofemoral joint earlier in life. We take as our starting point the investigation of whether PFOA is associated with previous anterior knee pain (AKP) in youth.

The classification and terminology surrounding patellofemoral disorders is contentious. "Anterior knee pain" is a broad symptom classification and a term about which there is arguably some agreement and acceptance among clinical researchers. It does not imply any particular diagnosis or physical condition and is likely to be multifactorial [2]. One Finnish study estimated the prevalence of AKP in childhood and adolescence at 19% [3], although prevalence estimates generally cited in the published literature vary widely, from 3% to 40% [4]. In such age groups AKP has traditionally been viewed as benign and self-limiting [5]. However, there is an increasing body of evidence to challenge this view. Stathopulu & Baildam [6] and Price et al. [7] both found that in excess of 90% of the AKP sufferers in their studies had ongoing problems at least four years later, while Sandow & Goodfellow reported that 94% of their sample continued to experience difficulties for a mean 16 years following diagnosis [5]. These studies do not provide evidence of a link between AKP in youth and the subsequent development of PFOA but they do highlight that AKP may have longer-term repercussions than previously thought.

Potentially plausible mechanisms linking AKP to PFOA include shared biomechanical risk factors, such as malalignment and muscular dysfunction. These are acknowledged risk factors for AKP that are also thought to be important in the pathogenesis of PFOA [8-10]. Other potential mechanisms include cumulative mechanical loading and micro-trauma and proprioceptive deficits. These remain relatively speculative. Our aim was not to test causal hypotheses but simply to systematically review the literature to describe the strength of evidence for a temporal association between AKP in younger adults and the subsequent development of PFOA. A clearly demonstrable association would provide impetus for further research on the underlying mechanisms with possible implications for the routine management of AKP problems and more widespread use of potential preventive strategies.

## Methods

### Eligibility criteria

Full-text articles in any language, identified via English titles and abstracts, dealing with in vivo human studies reported in international peer-reviewed journals were considered for inclusion. Foreign language articles were translated into English prior to review. To be included, studies had to contain quantitative data regarding structural changes indicative of PFOA (such as joint space narrowing, presence of osteophytes, subchondral sclerosis and cartilage defects), incident to original idiopathic AKP. Both prospective and retrospective designs were considered suitable for inclusion; cross-sectional studies and follow-up studies of patients who had undergone surgical interventions were not.

### Search strategy

Title and abstract searches were conducted in the following electronic databases from inception to December 2009: Pubmed, EMBASE, AMED, CINAHL, Cochrane, PEDro and SportDiscus. The following search terms were used for osteoarthritis: osteoarthritis, osteoarthrosis, arthritis, arthrosis, gonarthritis, gonarthrosis, total knee replacement, TKR, arthroplasty. Each of these terms was paired in turn with the following terms designed to capture all possible studies of anterior knee pain and related patellofemoral disorders: anterior knee pain, patellofemoral pain, patellofemoral arthropathy, chondromalacia patellae, jumpers knee. This strategy was supplemented by hand searching the reference lists of all full-text articles obtained and selected review articles. We cross-checked our search strategy by running additional searches in Pubmed using search terms from two published Cochrane systematic reviews of exercise therapy for patellofemoral pain syndrome [11] and knee osteoarthritis [12].

### Study selection

Two authors (MT and LW) independently reviewed all potentially relevant articles by title and abstract. Articles not excluded by both reviewers at this stage were obtained in full-text for detailed inspection. If agreement regarding inclusion could not be reached the fourth author (GP) acted as arbiter.

### Data extraction and synthesis

Data extraction included study location, design and date, sampling procedure and sample characteristics, AKP and PFOA definitions, follow-up duration, follow-up rate and main findings. Two authors (MT and LW) independently performed data extraction and then met to synthesise their findings. Disagreements were resolved via fourth author (GP) arbitration. Where possible within-study odds ratios or annual risk estimates were calculated to enable comparisons to be made between studies.

### Methodological quality

Studies included in the review were categorised using a conventional level of evidence hierarchy [13], which ranges from Level 1a (systematic reviews (with homogeneity) of randomised controlled trials) to Level 5 evidence (expert opinion without explicit critical appraisal, or based on physiology, bench research or "first principles"). In addition, two authors (MT and LW) independently employed the Critical Review Form - Quantitative Studies to assess the internal and external validity of included studies [14,15]. Using the 15 closed-ended questions, study quality was assessed by assigning "1" (indicating criterion completely fulfilled) or "0" (indicating criterion not fulfilled) to each question. These were then summed to generate a total score out of 15 [16,17]. Disagreements between the two reviewers were resolved via fourth author (GP) arbitration.

## Results

### Study characteristics

**Figure 1:**
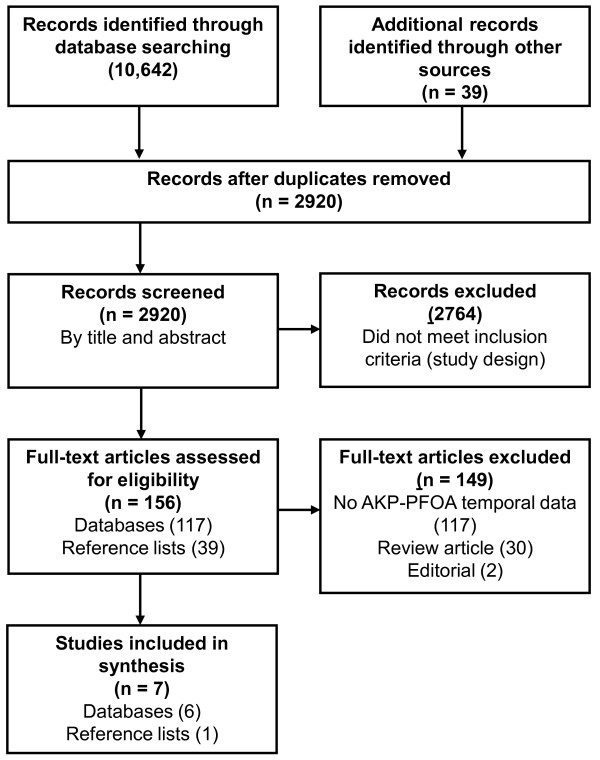
**Flow diagram summarising systematic search and process for selection of studies**.

The initial search identified 2920 potentially relevant articles, of which 2764 were excluded based on title and abstract. Full texts of 156 articles were independently reviewed by two authors (see Figure 1). Seven studies met all the inclusion criteria [18-24], one of these emerging from reference list screening [24]. Five studies were in English [18-20,22,24], and two were in German [21,23]. The design characteristics of the seven studies are presented in Table 1[25-27]. The cross-checking search identified five more potentially eligible studies which, on inspection of their full text, provided no quantitative data relevant to the present research question.

**Table 1:** Design characteristics of included studies

Author, year of publication	Design	Setting	Sampling procedure	AKP method of ascertainment, definition	PFOA method of ascertainment, definition	Length of follow-up
Utting et al 2005 [18]	Retrospective unmatched case-control.	Hospital orthopaedic surgery (Bristol, UK).	Local arthroplasty database (?period). Cases Isolated PF arthroplasty for severe isolated PFOA. Controls medial TF unicompartmental arthroplasty.	Self-complete questionnaire. Recall of AKP as a child, teenager, or young adult.	Severe isolated PFOA requiring patellofemoral arthroplasty.	-

Kannus et al 1999 [19]	Prospective follow-up of RCT.	Hospital orthopaedic outpatients (Tampere, Finland).	Consecutive patients with unilateral PFPS enrolled in RCT of conservative therapy (1987-1990).	Clinical interview, examination and plain x-ray. Retropatellar pain and crepitus with no other knee abnormalities.	Plain x-rays (AP, lateral, tunnel, tangential) and MRI. PFOA classified as none, mild, moderate, or severe for each imaging modality.	Mean 6.6 years.

Nimon et al 1998 [20]	Prospective follow-up of case series.	Hospital orthopaedic outpatients (Oxford, UK).	Consecutive adolescent female patients with idiopathic AKP (1974-1980).	Clinical history, examination, plain x-ray. No specific definition provided (other definitive diagnoses excluded, all complained of pain on ascending/descending stairs, squatting, or prolonged sitting).	Plain x-rays (AP, lateral, skyline). No specific definition provided.	Mean 16 years (range 14-20).

Imhoff & Boni 1989 [21]	Prospective follow-up of two case series.	Orthopaedic outpatients, (Zurich, Switzerland).	?consecutive patients with PF pain undergoing conservative or surgical treatment (1953-1968).	Unclear. No specific definition provided.	Plain x-rays (views not specified). Films scored using a 4-point radiographic definition, based on that of Jäger & Wirth [25].	Mean 23 years.

Hvid et al 1981 [22]	Prospective follow-up of case series.	Hospital orthopaedics/diagnostic radiology (Nykøbing, Denmark).	Patients with chondromalacia patellae on arthrography (1972-1977).	Clinical history, physical examination, and arthrogram. No specific definition provided. "a significant clinical syndrome" of chondromalacia patellae.	Plain x-rays (frontal, lateral, axial [26]). No specific definition provided ("signs of arthrosis were searched for on all films")	Mean 5.7 years.

Dexel et al 1980 [23]	Prospective follow-up of two case series.	Orthopaedic outpatients, (Zurich, Switzerland).	?consecutive patients with PF pain undergoing conservative or surgical treatment (1953-1968).	Unclear. No specific definition provided.	Plain x-rays (views not specified). 4-point radiographic definition, based on that of Tapper & Hoover [27].	Mean 13 years.

Karlson 1939 [24]	Prospective follow-up of comparative case series.	Hospital orthopaedics (Stockholm, Sweden).	? consecutive male patients (mostly army conscripts) with "simple chondromalacia patellae" of both traumatic and non-traumatic origin (1917-1934).	Clinical history and physical examination. No specific definition provided.	Plain x-rays (views not specified). No specific definition provided ("arthritis deformans").	Mean 5.9 years (range 1-20).

AKP Anterior knee pain; AP Anteroposterior; PF Patellofemoral; PFOA Patellofemoral osteoarthritis; PFPS Patellofemoral pain syndrome; RCT Randomised controlled trial; TF Tibiofemoral

One case-control study provided level 3b evidence [18]; the other six provided level 4 evidence, being prospective follow-ups of case series [19-24], one of participants in a randomised controlled trial (RCT) [19]. Three of the six prospective studies compared the long-term outcomes of participants with conservatively treated AKP versus those with operatively managed AKP [21,23,24]. For these three, only the data regarding the conservatively treated participants are reported here. The results of the studies' methodological quality assessment, according to Law et al.'s [14] Critical Review Form - Quantitative Studies, is presented in Table 2. Only four of the seven studies scored 8 or above out of the total possible score of 15 [18-20,22]. The other three scored between 3 and 5 [21,23,24], suggesting poor methodological quality. In particular, in only three out of the seven studies was the sample described in detail [18,22,23] and in only three were any limitations of the study acknowledged [18,19,22]. Definitions of AKP depended on clinical history and physical examination in four of the seven studies [19,20,22,24], but in only two of the seven was a specific definition of AKP provided [18,19], one of these being the recall of AKP as a child, teenager, or young adult [18].

**Table 2:** Methodological quality assessment

Quality criteria	**Utting et al 2005 **[18]	**Kannus et al 1999 **[19]	**Nimon et al 1998 **[20]	**Imhoff & Boni 1989 **[21]	**Hvid et al 1981 **[22]	**Dexel et al 1980 **[23]	**Karlson 1939 **[24]
Purpose clearly stated	1	1	1	0	1	0	0
Literature review relevant	1	1	1	1	1	0	1
Study design appropriate to study aims	1	0	1	1	1	1	1
No bias present	0	0	0	0	0	0	0
Sample described in detail	1	0	0	0	1	1	0
Sample size justified	0	0	0	0	0	0	0
Informed consent gained	0	0	0	0	0	0	0
Valid outcome measures used	0	0	0	0	1	0	0
Reliable outcome measures used	0	0	0	0	0	0	0
Results reported in terms of statistical significance	1	1	1	0	1	0	0
Analysis appropriate	1	1	1	1	1	1	1
Clinical importance of results reported	1	1	1	1	1	1	0
Appropriate conclusions	1	1	1	0	1	0	0
Clinical implications reported	0	1	1	1	1	1	0
Limitations of study acknowledged	1	1	0	0	1	0	0

**Total**	**9**	**8**	**8**	**5**	**11**	**5**	**3**

1, criterion fulfilled; 0, criterion not fulfilled.

### Study results

The results of the seven studies are summarised in Table 3. Only the retrospective case-control study had as its explicitly stated aim the investigation of a link between idiopathic adolescent AKP and PFOA [18]. This study compared the recall of adolescent AKP among 118 cases who had undergone patellofemoral arthroplasty for isolated PFOA, with an unmatched group of 116 'controls' who had undergone medial unicompartmental knee arthroplasty. Compared with controls, cases were more likely to report AKP during adolescence (22% versus 6%, crude OR 4.4: 95%CI 1.8, 10.6).

**Table 3:** Summary of results of included studies

Author, year of publication	N	Follow-up rate	Participant characteristics at baseline	Association between AKP and PFOA	Other findings	Quality score (0-15)
Utting et al 2005 [18]	234 (118 cases, 116 controls).	Response rate 78% (cases 79%, controls 77%).	Cases 90%F mean age at time of arthroplasty 67.3 years (range 44-87). Controls: 82%F mean age at time of arthroplasty 68.3 years (range 48-86).	Crude OR = 4.4 (95%CI 1.8, 10.6)	Prior history of AKP (22% vs 6%), PF instability (14% vs 1%), and patella trauma (16% vs 6%) among cases. No difference in mean age at onset of AKP (18.1 vs 19.4 years).	9

Kannus et al 1999 [19]	37	70%	Based on 49 potentially eligible participants: 53% F Mean age 27 years (range 15-50). Mean duration of symptoms 16 months (SD 19).	-	PFOA at follow-up (mean 6.6 years). On plain x-ray: mild 16%, moderate 0%, severe 3% On MRI: mild 11%, moderate 19%, severe 5%. Estimated annual risk of PFOA 2.9%.	8

Nimon et al 1998 [20]	8	13%	Based on 63 potentially eligible patients: 100%F Mean age 15.5 years (range 10-19).	-	Plain x-rays "normal" on participants imaged at follow-up (mean 16 years). Estimated annual risk of PFOA 0%.	8

Imhoff & Boni 1989 [21]	29	19%	55%F Mean age at diagnosis 23 years (range 7-52). 11 had a history of knee trauma.	-	PFOA at follow-up (mean 23 years): Small superior patellar osteophytes (31%), joint space narrowing/ ↑ osteophytes/subchondral sclerosis 10.3%, joint space obliteration/cystic changes 6.9%. Estimated annual risk of PFOA 2.1%.	5

Hvid et al 1981 [22]	22	100% (?)	60%F Mean age 27 years (range 14-44). Mean duration of symptoms 7.6 years (range 4-16).	-	No arthrosis in any participant at follow-up (mean 5.7 years). Estimated annual risk of PFOA 0%.	11

Dexel et al 1980 [23]	25	15%	32%F Mean age 25 years (range 15-52). 5 had a history of post-traumatic patellar chondropathy.	-	Moderate or severe changes consistent with PFOA at follow-up (mean 13 years) 12%. Estimated annual risk of PFOA 0.9%.	5

Karlson 1939 [24]	35	31%	Based on 71 potential eligible patients: 0%F 11% aged <20 years, 80% 20-29, 8% 30-59.	-	"very slight" arthritis deformans at follow-up (mean 5.9 years) 20%. Estimated annual risk of PFOA 3.4%.	3

AKP Anterior knee pain; F Female; OR Odds ratio; PF patellofemoral; PFOA Patellofemoral osteoarthritis; 95%CI 95 percent confidence interval

The study by Kannus et al [19] - a 7-year follow-up of participants in an RCT of non-operative treatments for patellofemoral pain syndrome, reported a 70% follow-up rate of participants who received imaging at baseline (37 out of 53). Plain radiographs and MRI scans were taken of the patellofemoral joints of all 37 participants. Although some degree of structural abnormality consistent with PFOA changes was found to be present in 13 of the 37 (35%) using MRI, in only two cases (5%) were these changes deemed to be indicative of severe PFOA. A similar prevalence for severe PFOA was observed for x-rays (3%). Of the five long-term follow-ups of case series, plain x-rays of the patellofemoral joint were available in varying proportions of the samples [20-24]. Four of the five reported follow-up radiographic data on less than a third of the original sample [20,21,23,24]. Two studies reported no radiographic changes [20,22], while three documented changes consistent with PFOA in 12%, 20% and 48% of cases at 13, 5.9 and 23 years of follow-up, respectively [23,24,21].

With the exception of the case-control study by Utting et al. [18] the number of participants in the studies was low, generally numbering between 22 and 37 (although one study did only have eight participants [20]). Follow-up rates were also often poor because of the intervening time-lag: three of the studies had follow-up rates of less than 20% [20,21,23].

## Discussion

These findings reveal a paucity of high-quality research evidence regarding the link between idiopathic AKP in younger adults and the subsequent development of PFOA. The results of Utting et al.'s [18] case-control study provide some evidence for this link. While the accuracy of individuals' recall is an issue in such studies, the choice of a suitable control group (comprising individuals undergoing medial unicompartmental arthroplasty) is likely to have minimised recall bias.

The results of the remaining six studies should be interpreted with caution for a number of reasons, including issues of small sample size, poor follow-up rates, absence of control groups and samples containing individuals with traumatic AKP. It should be noted that in some cases studies were so selective about which participants they chose to follow up with x-ray investigations that their use in evaluating the existence of a link between idiopathic AKP and subsequent PFOA is questionable. Hvid et al. [22] only followed up individuals with "a significant clinical syndrome" of chondromalacia patellae confirmed on arthrograms, possibly suggesting pre-existing patellofemoral arthropathy, while participants invited for x-ray follow-up by Nimon et al. [20] were only that small proportion of 14 out of 49 whose symptoms had got no better over the years. Furthermore, in five studies either the imaging views are not specified [21,23,24] or the criteria by which PFOA was judged to be present or absent are not stated [20,22,24]. Additional limitations include the lack of substantive definitions of AKP and PFOA in many cases. The unclear clinical significance of mild structural abnormalities, for example the 35% 7-year incidence of any MRI changes in the study by Kannus et al. [19], also requires cautious interpretation, particularly in the absence of control groups with no AKP at the time of recruitment.

Agreed quality checklists, such as that used in the current review, can provide a profile of a research study report that draws attention to its particular methodological strengths and weaknesses [28]. Most of the studies in this review failed to satisfy several of the methodological quality criteria in this checklist. This is partly due to the fact that such tools necessarily tend to reflect the quality of reporting of data relevant to a clear principal research question. Most of the studies included in this review either did not have a clear research question beyond a general descriptive epidemiological remit, or they reported data regarding AKP and PFOA that was incidental to their main purpose.

The studies identified in this review underscore the major challenges in designing and conducting prospective studies to answer the question of whether there may be an association between AKP in younger adulthood and the development of PFOA later in life. These are 1) recruiting a representative sample, 2) using standardised definitions of AKP and PFOA to both an exposure group and a comparable control group, 3) performing x-rays at baseline to confirm the absence of PFOA in AKP sufferers at this stage 4) ensuring adequate follow-up and low attrition over a long period. While such studies as those identified in this review can hint at the possibility of an association between AKP in younger adults and the subsequent development of PFOA in later life, a clear causal relationship cannot be established by them.

Several limitations in our study deserve comment. Firstly, the majority of studies identified by this review did not directly investigate the link between AKP and PFOA. It is therefore possible that some studies excluded by title and abstract search could also contain additional data without having given explicit focus to this question. Secondly, we did not formally attempt to quantify the level of agreement between reviewers either in the selection of articles for inclusion or in the methodological quality assessment.

## Conclusions

In summary, there is a lack of sound evidence from epidemiological studies on the association between AKP in younger adults and subsequent PFOA. What evidence there is comes from one case-control study. Much of the other 'evidence' relating to this research question is tentative, exploratory, even incidental to the authors' main objectives, and is therefore still subject to confirmation or falsification with less biased study designs in larger samples. The possibility that AKP is a risk factor for incident PFOA warrants further attention. Future well-designed analyses, conducted within population-based studies are needed. It is possible that existing longitudinal studies with actual or potential AKP and PFOA data may be capable of addressing this question.

## Competing interests

The authors declare that they have no competing interests.

## Authors' contributions

All authors participated in the study design and analysis, and drafting of the manuscript. All authors read and approved the final manuscript.

## Pre-publication history

The pre-publication history for this paper can be accessed here:

http://www.biomedcentral.com/1471-2474/11/201/prepub
